# Left Ventricular Deformation and Rotational Mechanics in Various Pathologies—The Role of the Pattern of Abnormalities

**DOI:** 10.3390/jcm12082840

**Published:** 2023-04-13

**Authors:** Attila Nemes

**Affiliations:** Department of Medicine, Albert Szent-Györgyi Medical School, University of Szeged, 6725 Szeged, Hungary; nemes.attila@med.u-szeged.hu; Tel.: +36-62-545220; Fax: +36-62-544568

Non-invasive assessment of myocardial mechanics using modern imaging techniques became a current topic due to the rapid developments in echocardiography and cardiac magnetic resonance imaging (cMRI) [1,2,3,4,5,6,7,8,9]. During the cardiac cycle, the walls of the left ventricle (LV) not only contract, but simultaneously rotate displaying a spatial movement pattern. Previous studies raised the possibility of disease-specific abnormalities of myocardial mechanics in certain disorders [1,2,3,4,5,6,7,8,9].

According to recent guidelines, the characteristics of LV rotational mechanics can be assessed by three-dimensional (3D) speckle-tracking echocardiography (STE) due to their 3D nature [1]. This method allows the concurrent quantification of basal and apical LV rotations and left atrial (LA) measurements, allowing for (patho)physiological assessments. In a recent study, dependence of 3DSTE-derived LV rotations on LA volume changes throughout the cardiac cycle was investigated in normal physiological conditions. A detailed analysis showed strong associations between 3DSTE-derived LA volumes and LV rotational mechanics: systolic basal LV rotation was the highest in the case of the highest systolic maximum LA volume and early diastolic pre-atrial contraction LA volume. Apical LV rotation did not show obvious relationships with increased LA volumes. The highest systolic basal LV rotation was associated with significantly increased diastolic LA volumes, while reduced diastolic LA volumes were observed in cases of increased apical LV rotation. These results highlight a strong relationship between LA volumes and LV rotational mechanics even in normal physiological conditions [1].

Some new theories suggested the possibility that LV also rotates longitudinally during the cardiac cycle. Moreover, abnormalities in patients with complete left bundle branch block (cLBBB) were suggested to be due to a related ventricular dyssynchrony and reduction in systolic and diastolic efficiencies. In a recent two-dimensional (2D) STE study, patients with normal QRS, complete right bundle branch block (cRBBB) and cLBBB were compared to clarify any differences in spatial LV motions. All patients with cLBBB showed significantly a larger clockwise longitudinal rotation than patients with normal QRS or cRBBB. This longitudinal rotation correlated with QRS duration, but was not associated with low LV ejection fraction (EF) or the presence of regional wall abnormalities [2].

In normal circumstances, there is a complex contractility pattern, which can be quantified by STE. The myocardial shortening from LV base to LV apex is determined by global longitudinal strain (GLS), the systolic LV shortening in short-axis views is quantified by global circumferential strain (GCS) and the myocardial thickening from the endocardium to epicardium is measured as global radial strain (GRS). Similar parameters could be measured for the right ventricle (RV) and atria as well [1,2,3,4,5,6,7,8,9].

There is inconsistent agreement between 2DSTE- and 3DSTE-derived LV-GLS, when assessed in different clinical scenarios, e.g., in patients with normal LV, reduced LV-EF and cardiac pacing. According to the study of Plasek et al., LV-GLS assessed by 2DSTE and 3DSTE is closely associated, but only on a global level suggesting that LV-GLS comparisons are more representative of global shortening than local displacement. When the effects of RV pacing and decreased LV-EF were investigated, a reduced correlation between 2DSTE- vs. 3DSTE-derived LV-GLS was observed [3].

Strain measurements allow detailed analyses of LV contractility, which can be of particular importance in the assessment of cardiomyopathies. In hypertrophic cardiomyopathy (HCM), LV-GLS is known to be impaired and is associated with an increased risk. In a recent study, the analysis of 2DSTE-derived LV-GLS adjusted to the regional thickness allowed better evaluation of myocardial deformation in HCM, especially in the most hypertrophic LV segments [4]. Moreover, use of disopyramide, a sodium-channel blocker classified as a class 1a agent, was associated with significant reduction of 2DSTE-derived LV-GLS with preserved LV-EF and an unchanged outflow gradient demonstrating its acute negative inotropic effect on the myocardium in patients with obstructive HCM [5]. 

LV noncompaction cardiomyopathy (LVNC) is a relatively new clinical entity characterized by prominent trabecular LV meshwork and deep intertrabecular recesses. When LV noncompaction was diagnosed in pediatric patients by cMRI, LV enlargement and reduced LV systolic function (lower LV strains and LV-EF) were found together with increased RV trabeculations and subclinical impairment of RV myocardial deformation (decreased RV-GRS and RV-GCS with preserved RV-EF) confirming the involvement of the left and right heart in varying degrees [6]. 

Fabry disease (FD) is an inherited, X-linked, rare, lysosomal storage disease, affecting the heart among other organs. It is also known as alpha-galactosidase-A deficiency leading to the accumulation of sphingolipids in certain organs. In a recent cMRI study, LV-GRS and LV-GLS were found to be reduced in FD patients; moreover, LV-GLS also showed correlations with the FD-specific biomarker globotriaosylsphingosine (LysoGb3) [7]. These findings could suggest early signs of cardiac involvement in FD patients.

Chagas disease (CD) is a tropical vector-borne infection caused by the protozoan parasite Trypanosoma cruzi. Chagas cardiomyopathy (CM) is the leading cause of non-ischemic cardiomyopathy with diffuse myocarditis and focal fibrosis in Latin America. In a recent meta-analysis, different levels of functional derangements in myocardial function across different stages of CD were found. CM patients had a significantly higher LV-GLS compared to patients with the indeterminate form (IF) of CD, while differences between IF patients and healthy controls could not be detected. Segmental strain analyses revealed that patients with the CD-IF form had worse strain values in the basal-inferoseptal and mid-inferoseptal segments compared to healthy controls [8]. 

Finally, LV and LA myocardial deformation was examined in patients following acute myocardial infarction. The presence of chronic obstructive pulmonary disease was associated with more impaired 2DSTE-derived LV and LA functions, which was independent of age, LV-EF and pulmonary function test parameters [9].

All these findings strengthen previous results confirming the importance of LV rotational and deformation parameters calculated during strain analysis in various pathologies. In addition to the above, the possibility of the significance of disease-specific differences may also arise. However, studying the prognostic impact of these variables is still a task for future investigations in the relevant disorders. 

## Data Availability

All information are available in cited papers published in the Journal of Clinical Medicine.
